# Evaluation of Pain, Dietary Intake, Body Mass Index, and Periodontal Status in Patients Undergoing Fixed Orthodontic Treatment With Bite Raiser

**DOI:** 10.7759/cureus.32800

**Published:** 2022-12-21

**Authors:** Majid Shalchi, Marjan Mahdavi Roshan, Mohammad Moslem Imani, Reihaneh Aghajani, Samar Khabbaz, Elaheh Shafiei Haghshenas

**Affiliations:** 1 Orthodontic Department, Guilan University of Medical Sciences, School of Dentistry, Rasht, IRN; 2 Clinical Nutrition Department, Guilan University of Medical Sciences, School of Medicine, Rasht, IRN; 3 Orthodontic Department, Kermanshah University of Medical Sciences, School of Dentistry, Kermanshah, IRN; 4 Dental Research Center, Shahid Beheshti University of Medical Sciences, Tehran, IRN; 5 Orthodontic Department, Howard University Collage of Dentistry, Washington, USA

**Keywords:** periodontal index, body mass index, nutrient intake, visual analog scale, fixed appliances, orthodontic bite-raiser

## Abstract

Background

The aim of this study is to evaluate the effect of bite raisers on patients’ body mass index (BMI), nutrient intake, periodontal status, and pain experienced during orthodontic treatment.

Material and Methods

This cohort study included 44 patients aged 18 to 35 years old; undergoing fixed orthodontic treatment. Patients of the intervention group received a composite resin bite raiser (3M Espe, St. Paul, USA) over their first mandibular molars along with fixed orthodontic appliances (slot 0.022 × 0.030”, 3M Unitek, Monrovia, Calif), while the control group only received a fixed orthodontic appliance. Pain levels using the visual analog scale (VAS), dietary intake (calories, proteins, carbohydrate, and fat intake), body mass index (BMI), tooth mobility, bleeding on probing (BOP), and pocket depth (PD) were assessed for all patients in the first three months after fixed orthodontic appliance placement. The data were analyzed using SPSS software version 22.0 (IBM Corp, Armonk, NY, USA) at a significance level of 0.05.

Results

The pain had an increasing and then decreasing significant trend during the study for all patients (P<0.001). Calory intake also had an increasing and then decreasing significant trend for all participants (P=0.007). The consumption of carbohydrates and BMI significantly decreased during the study in both groups of patients (P<0.01) and tooth mobility, BOP, and PD significantly increased for all participants (P<0.001). No significant differences were observed between the intervention and control groups in terms of the above-mentioned variables.

Conclusion

The application of bite raiser does not influence patients’ pain, dietary intake, BMI, and periodontal status. However, fixed orthodontic appliances affect patients’ calorie and carbohydrate intake, patients’ BMI, and periodontal indexes including tooth mobility, BOP, and PD.

## Introduction

Temporary bite raisers, also known as bite ramps, bite, pillow bite turbos, or speed pumps, are commonly required during orthodontic procedures when treating patients with anterior crossbites, posterior crossbites, deep bites, and scissor bites. Bite-raisers are used to facilitate tooth movement and prevent traumatic occlusion and bracket shear. Posterior bite raisers intrude the maxillary and mandibular molars, while anterior bite raisers intrude the anterior teeth and extrude the posterior dentition [1,2]. Although there are different types of bite raisers including removable posterior bite plates, bonded lingual plates, bonded occlusal build-ups, Gray bite raisers, and temporary bite opening crowns, most patients prefer bonded bite raisers since they are more convenient [3,4].

It has been reported that orthodontic treatments affect patients’ oral health, quality of life, eating habits, and chewing inability [5-7]. There are some studies showing that patients usually prefer soft food during their orthodontic treatment to decrease their pain and discomfort when eating, and this can influence the nutritional value and the quality of their consumed foods [8-11]. Most patients undergo orthodontic treatments during their adolescence. Thus, the importance of nutritional needs and their effects on patients’ growth, development, and maturity is very critical [10,11]. Therefore, orthodontists are encouraged to attribute special attention to patients’ diets during orthodontic treatment [5,11,12].

Bite-raisers also affect the surrounding periodontium of the teeth that they are bonded to. As we know, the anatomy of these bonded teeth will temporarily change after bite-raiser placement, which leads to the concentration of masticatory forces on them, and this increased load may affect the function and health of the surrounding periodontium [7,13].

The aim of this study is to evaluate the effect of bite raisers on patients’ body mass index (BMI), nutrient intake, periodontal status, and pain experienced during orthodontic treatment.

## Materials and methods

This cohort study was approved by the Ethics Committee of the Dental Research Center at Guilan University of Medical Science, Rasht, Iran. The study included 44 patients aged 18 to 35 years old referred to the orthodontic department of the Guilan University of Medical Science, School of Dentistry. Participating patients were candidates for fixed orthodontic treatment, with no craniofacial deformity and no history of trauma. Patients with a history of fixed orthodontic appliances, smoking, and systemic disease were excluded. The other exclusion criteria include taking medicine affecting the oral cavity, within the last three months, having a periodontal pocket depth of more than 3 mm, and having active dental caries. Neither NiTi nor rectangular wires were used during the three months of the present study, and no patient needed extraction for their orthodontic treatment. 

Twenty-two participants received fixed orthodontic appliances (slot 0.022 × 0.030”, 3M Unitek, Monrovia, Calif) and 2-4 mm composite resin bite raiser (3M Espe, St. Paul, USA) over their first mandibular molars (intervention group), and the other 22 patients only received fixed orthodontic appliances without bite raiser (control group). 

Patients’ pain and discomfort were assessed using the visual analog scale (VAS) at baseline, and then every hour for six hours after appliance placement. Pain assessment was also continued once a day, for the next seven days. The pain was recorded in the range of 0 (no pain) to 10 (unbearable pain). Any analgesics consumption or disturbance in daily activities (chewing, speaking, etc.) has been recorded. 

Participants’ dietary intake (calories, proteins, carbohydrate, and fat intake) and BMI were recorded at baseline, one month, and three months after the placement of the fixed orthodontic appliances. Dietary intake was assessed using a 24-hour memory recall and was analyzed using Nutrition software version 5.3 (First Data Bank, San Bruno, CA, USA). Weight was measured using a digital scale (POCFGST, China) and was reported in kg with 0.1 standard error. Height was measured with a wall-mounted Harpenden stadiometer (Detecto, MI, USA) and was reported in cm with 0.1 standard error. BMI was calculated as weight/height2 and was reported as kg/m².

Tooth mobility, bleeding on probing (BOP) and pocket depth (PD) were also evaluated at baseline, one month, and three months after the placement of the fixed orthodontic appliances. Mobility was reported as score 0 (0.1 to 0.2 mm mobility; physiological mobility), score 1 (1 mm horizontal mobility), score 2 (more than 1 mm horizontal mobility), and score 3 (vertical and horizontal mobility with pain in function). To assess the BOP, William’s probe (CP-12/thin Williams color-coded probe, Hu-Friedy, Chicago, IL, USA) was moved in the subgingival zone around the first molar of each quadrant; after 30 seconds the presence of bleeding was considered as positive BOP. Moreover, PD was measured in the mid-buccal, mid-lingual, mesio-buccal, and disto-buccal points around the first molar of each quadrant, and then the mean of PD was reported for each patient. 

The data were analyzed using SPSS software version 22.0 (IBM Corp, Armonk, NY, USA). Independent t-test, repeated measures, Greenhouse-Geisser, Huynh-Feldt, and Bonferroni tests were applied at the significance level of 0.05. 

## Results

No patient was excluded during the study. 56.5% (13) of participants in the intervention group (Patients with Bite-raisers) were male and 42.9% (9) were female. 43.5% (10) of participants in the control group (patients without Bite-Raisers) were male and 57.1% (12) were female. The mean age of patients in the intervention and control groups was 24.27±3.25 and 24.36±3.93 respectively. Therefore, the participants were homogeneously distributed in terms of age and gender. No patient reported analgesics consumption or disturbance in daily activities. 

**Figure 1:**
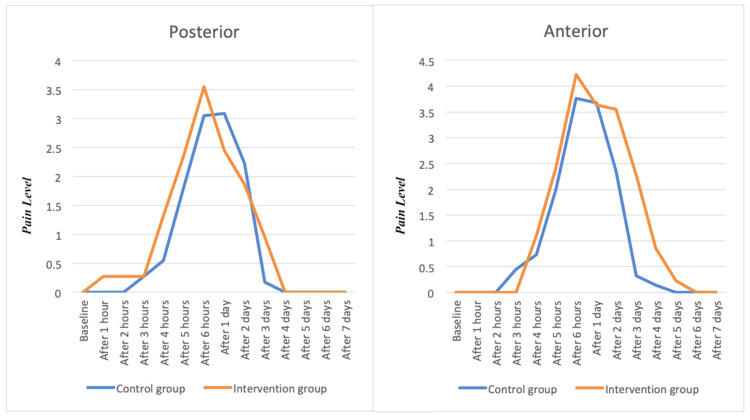
This chart demonstrates the pain level in two groups of patients (intervention group with bite raiser and control group without bite raiser) in the first 7 days after placement of appliances.

The reported pain by patients in both groups had an increasing and then decreasing significant trend during the study (P<0.001). The pain was higher in the first two to three days for all patients and then it decreased. The pain level was also significantly higher on the anterior teeth compared to the posterior dentition (P<0.001). There was no significant difference between patients’ pain levels in the two different groups. (Figure 1)

The changes in the diet of all patients were assessed using calories, proteins, carbohydrates, and fat intake. Results indicated that the calorie intake had an increasing and then decreasing significant trend for all patients (P=0.007). Moreover, only carbohydrate intake was significantly increased in all patients (P=0.008), and consumption of fat and protein was not significantly changed.

There was no significant difference in using calories, proteins, carbohydrates, and fat intake between patients with bite-raisers (intervention group) and without bite-raisers (control group) (Table 1).

**Table 1:** The mean of calories, protein, carbohydrate, and fat intake according to the study groups at baseline, 1- and 3- months after orthodontic appliance placement.

		Baseline	1 month after appliance placement	3 months after appliance placement
Calories (Kcal)	Intervention group	1130.20±625.74	1594.50±418.41	1571.51±426.75
		(P>0.05)	(P>0.05)	(P>0.05)
	Control group	1272.79±462.21	1740.81±798.07	1627.17±712.68
Protein (gr)	Intervention group	68.96±38.23	70.78±19.34	62.46±14.14
		(P>0.05)	(P>0.05)	(P>0.05)
	Control group	65.11±34.91	79.01±26.72	75.26±26.72
Carbohydrate (gr)	Intervention group	171.30±80.74	197.29±68.19	206.90±70.89
		(P>0.05)	(P>0.05)	(P>0.05)
	Control group	149.30±68.08	223.22±118.02	228.27±102.50
Fat (gr)	Intervention group	49.14±28.65	54.30±23.23	51.10±22.29
		(P>0.05)	(P>0.05)	(P>0.05)
	Control group	43.96±26.94	55.54±33.96	55.86±29.43

Assessment of patients’ BMI also showed that BMI was significantly decreased in all participants (P<0.001). However, no significant difference was observed between the patients in the intervention and control groups in terms of BMI (P=0.648) (Table 2).

**Table 2:** The mean body mass index of patients in the two study groups at baseline, 1, and 3 months after orthodontic appliance placement.

Body Mass Index	Baseline	1 month after appliance placement	3 months after appliance placement
Intervention group	23.10±1.62	22.64±1.71	22.34±1.64
	(P>0.05)	(P>0.05)	(P>0.05)
Control group	23.44±2.43	22.98±2.45	22.73±2.45

The tooth mobility, BOP, and PD were also assessed in both study groups. Findings indicated that all three factors significantly increased in both intervention and control groups during the study (P<0.001). But these indexes were not significantly different between the patients in the intervention group and the control group (Table 3).

**Table 3:** The mean of tooth mobility, bleeding on probing, and pocket depth according to the study groups at baseline, 1, and 3 months after orthodontic appliance placement.

		Baseline	1 month after appliance placement	3 months after appliance placement
Tooth Mobility	Intervention group	0±0	0.79±0.40	0.95±0.21
		(P>0.05)	(P>0.05)	(P>0.05)
	Control group	0±0	0.77±0.41	1±0
Bleeding on Probing	Intervention group	0.04±0.06	0.16±0.15	0.28±0.16
		(P>0.05)	(P>0.05)	(P>0.05)
	Control group	0.08±0.13	0.2±0.21	0.30±0.21
Pocket Depth	Intervention group	1.41±0.20	1.88±0.24	2.12±0.26
		(P>0.05)	(P>0.05)	(P>0.05)
	Control group	1.430.32	1.89±0.42	2.15±0.32

## Discussion

This study was performed on 44 patients satisfying the inclusion criteria of the study. Fifty percent (22) of participants received bite-raisers as part of their orthodontic treatment and 50% (22) did not. In the current study, no patients reported disturbance in their daily activities due to the orthodontic treatment, which was similar to the findings of Erdinç et al. [14].

In this study, patients experienced increasing pain during the first six hours around the anterior and posterior teeth in both groups. Six hours after the placement of the fixed orthodontic appliance, the pain had a decreasing trend. Similarly, Grewal et al. [15], Shenoy et al. [16], Eslamian et al. [17], and Topolski et al. [18] reported that the peak of pain was experienced six hours after the beginning of the orthodontic treatment.

According to the results of the current study, participants of both study groups experienced higher pain around the anterior teeth compared to their posterior dentition. This finding is in accordance with the findings of Erdinç et al. [14]. The posterior teeth are less involved during the leveling phase of orthodontic treatment, and they also have more root surface, more periodontium surface, and more force distribution. Therefore, it is rational that patients reported higher levels of pain around the anterior teeth compared to the posterior ones. 

In the present study, the calorie intake increased during the first month and then decreased from the first to the third month after fixed orthodontic appliance placement. Moreover, only carbohydrate intake increased during the study, and fat and protein consumption were not changed. Ozdemir et al. [19], Shirazi et al. [9], and Riordan et al. [10] reported that the total daily calorie did not change during their study. Riordan et al. [10] reported that carbohydrate intake decreased after fixed orthodontic appliance placement. However, Ozdemir et al. [19], Shirazi et al. [9] did not report any change in carbohydrate and protein intake during the orthodontic treatment. Furthermore, Ozdemir et al. [19] stated that the intake of fat decreased during the first-week post-treatment and increased afterward. Shirazi et al. [9] reported that the intake of cholesterol, saturated fat, and monounsaturated fat significantly decreased during their study. Riordan et al. [10] and Sharma et al. [20] found that the intake of fat increased during orthodontic treatment. This contrary to the results may be due to the different eating cultures in these studies. 

The results of this study showed BMI significantly decreased in all patients, which is consistent with Sandeep et al. [8], Ajwa et al. [21], Schott et al. [22], Kılınç et al. [23] and Soni et al. [24]. During the orthodontic treatment, patients tend to consume soft foods more commonly to minimize the experienced pain and discomfort, and that causes a decrease in patients’ BMI, especially during the first three to six months after the beginning of the treatment.

All participants in the present study had increased tooth mobility, BOP, and PD after orthodontic appliance placement. Previous research [25] reported that orthodontic treatment might increase the development of gingivitis in patients. The authors stated that orthodontic brackets and elastics can interfere with effective dental plaque removal and that leads to increased plaque accumulation, which causes BOP and increased pocket depth. Moreover, orthodontic forces temporarily increase tooth mobility during the orthodontic treatment and this mobility is gradually restored to a standard level after the completion of the treatment [26]. 

## Conclusions

According to the findings of this study, the pain, dietary, BMI, and periodontal status of patients with bite-raisers were not significantly different from those of the control group who only had fixed orthodontic appliances with no bite-raisers. This means that bite-raisers have limited influence on the factors mentioned above.

Therefore, the application of bite raiser does not influence patients’ pain, dietary intake, BMI, and periodontal status. However, fixed orthodontic appliances affect patients’ calorie and carbohydrate intake, BMI, and periodontal indexes including tooth mobility, BOP, and PD.
